# Agronomic performance of lettuce cultivars submitted to different irrigation depths

**DOI:** 10.1371/journal.pone.0224264

**Published:** 2019-12-11

**Authors:** Claudinei Martins Guimarães, Fernando França da Cunha, Francisco Charles dos Santos Silva, Edcássio Dias Araújo, Aline Baldez Felismino Guimarães, Everardo Chartuni Mantovani, Derly José Henriques da Silva

**Affiliations:** 1 Department of Agricultural Engineering, Center of Agricultural Sciences, Federal University of Viçosa, Viçosa-MG, Brazil; 2 Department of Domestic Economy, Center of Human Sciences, Federal University of Viçosa, Viçosa-MG, Brazil; 3 Department of Phytotechnics, Center of Agricultural Sciences, Federal University of Viçosa, Viçosa-MG, Brazil; Institute for Horticultural Plants, China Agricultural University, CHINA

## Abstract

The use of cultivars adapted to the climate and soil conditions associated with adequate irrigation supply maximizes lettuce agronomic performance. The aim of this work was to evaluate the agronomic performance of four lettuce cultivars submitted to five different drip irrigation depths under a protected environment in Viçosa-MG, Brazil. A randomized block design was applied in a split plot scheme with four replications, and several agronomic characteristics were evaluated by analysis of variance, Tukey range tests, regression and principal component analysis. A higher chlorophyll concentration in the Raider Plus cultivar promoted the production of more leaves, leading to a higher phytomass. The Luara cultivar presented a higher number of commercial leaves per plant than the other cultivars, regardless of the irrigation depth, reflected in a larger diameter and volume of the aerial part of the plants. The Raider Plus and Luara cultivars presented better root development than that of the other cultivars, reducing the effect of plant water stress even under lower irrigation depth conditions. Although higher water productivity (WP) was observed for the lowest irrigation depth (50% of ETc), important variables reached the maximum values at depths higher than 100% water replenishment. Therefore, Raider Plus and Luara cultivars with an irrigation depth of 110% of crop evapotranspiration provided better commercial lettuce quality and are recommended for lettuce cultivation in the research region and under conditions similar to those of the present study.

## Introduction

The cultivated lettuce (*Lactuca sativa* L.) of the Asteraceae family is one of the most popular and consumed vegetables in the world [1, 2] and the main leafy vegetable marketed in Brazil [3]. Generally, it is consumed in salads and represents a good source of minerals [4]. Grown all over Brazil, traditionally on small family farms, lettuce has great economic and social importance [5], strengthening self-reliance and improving the subsistence of small farmers [6].

The choice of cultivars that are more adapted to the climate and soil type of a region allows an increase in crop yield. Thus, due to its origin of temperate climate, it was necessary to develop new lettuce cultivars that were more adapted to tropical conditions over the years [7]. In addition, each region of tropical climate has very specific local soil and climatic characteristics to be considered, which justifies the study of cultivars adapted to each region. Currently, the main types of lettuce grown in Brazil are crisp, American, smooth and others (red, Mimosa, and Roman, among others), corresponding to 70%, 15%, 10% and 5% of the Brazilian market, respectively [3].

Lettuce cultivation is usually performed in a protected environment or in the open field [8], but the latter does not include the quantification of the necessary irrigation depth [9]. Therefore, the use of a protected environment, considered intensive [10], has become more frequent because it protects crops from climatic adversities, pests and diseases, increasing the overall yield and product quality [11]. The use of irrigation enables successive crops throughout the year [9] and is obligatory in a protected environment to supply plant water needs.

Another important factor is an efficient irrigation method to fully or supplement the water needs of the crops. Second [12], the most commonly used method (conventional sprinkler) does not include aspects that are fundamental to the sustainability of irrigated agricultural activity, such as plant health and multiple uses of water. This fact favors the use of localized drip irrigation, successfully adopted for several crops, with great advantages, such as reduced energy consumption, facilitated and favored plant health control and greater efficiency in water productivity (WP).

Combined with an efficient irrigation method, proper irrigation water management enhances crop quality and productivity. Considering the intensive cultivation in a protected environment, the study of appropriate irrigation depths for each crop and region is even more important. According to [13], water is indispensable for production and therefore its lack or excess significantly affects the productivity of a crop, making its rational management indispensable to achieving the maximization of production.

In this sense, the aim of this work was to evaluate the agronomic performance of lettuce cultivars submitted to different drip irrigation depths, indicating suitable cultivars and soil water replacement depths for the cultivation of lettuce in a protected environment in the region of Viçosa-MG, Brazil.

## Materials and methods

### Experimental area

The present research was located at the Federal University of Viçosa (UFV)—Viçosa-MG, Brazil, at coordinates 20° 45' 14” S, 42° 52' 55” W and an altitude of 648 m above sea level (S1 Fig). The region climate of the type Cwb [14] presents an annual average temperature of 19.4°C and annual rainfall of approximately 1,200 mm.

### Installation and conduct of the research

**Fig 1:**
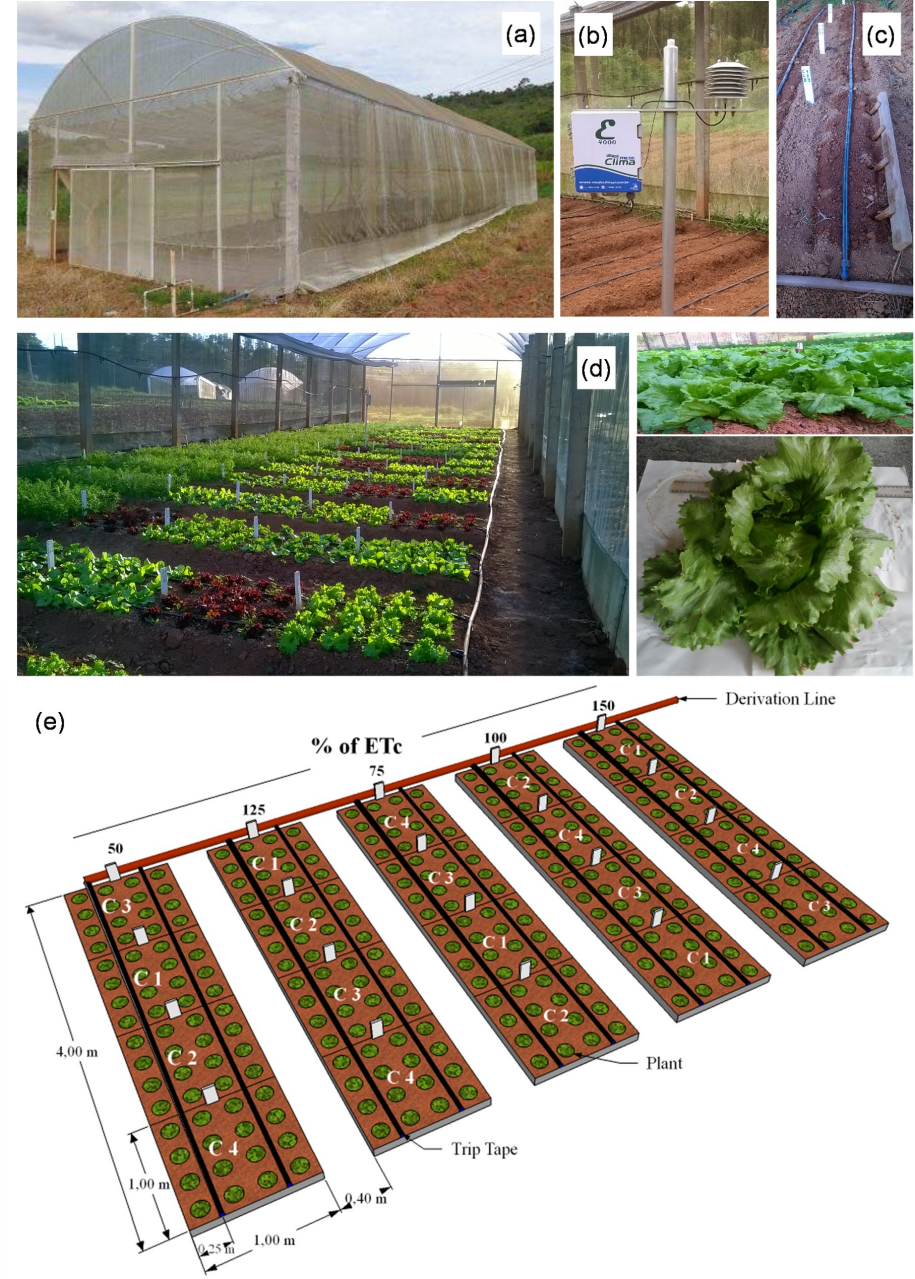
Research installation location (a, b, c and d) and structure of one of the four blocks (e). C: lettuce cultivars. Viçosa-MG, 2017.

A protected environment (Fig 1) with a total area of 240 m^2^ (8 m wide, 30 m long and right foot of 3.2 m) was used, with the sides covered by polyethylene wire mesh (100% polyethylene, 25 mesh—1.0 × 1.0 mm aperture, with 10 wires cm-1 screen, and 25% shading) and a roof constructed of blue plastic (AV Blue, 120 microns, 78% and 67% transmission and diffusion of light, respectively).

A eutrophic Red-Yellow Latosol (Table 1) was used. Soil preparation was carried out by plowing, harrowing and passing the enchanter. Chemical and physical water analyses (Table 1) were performed, and liming and chemical fertilization were performed based on the results of the soil chemical analysis and recommendations of [15].

**Table 1:** Soil physical-hydrological and chemical analyses, according to [16].

Depth	FC	PWP	Ds	Clay		Silt		Sand		Textural Classification second [18]			
	second [17]												
cm	g g^-1^		g cm^-3^	%									
0–20	0.45	0.27	1.23	39		11		49		Sandy-clay			
Depth	pH	P	K	Ca^2+^	MG^2+^	Al^3+^	H+Al	SB	CEC_(t)_	CEC_(T)_	V	m	P-rem
cm	H_2_O	mg dm^-3^		cmol_c_ dm^-3^							%		mg L^-1^
0–20	6.1	328.4	196.0	5.4	1.2	0.0	2.6	7.2	7.2	9.8	73.3	0.0	51.5

FC: moisture in field capacity (tension of 33 kPa); PWP: moisture at permanent wilting point (tension of 1,500 kPa); Ds: Soil density; Available P and K, extracted with Mehlich I; Ca, Mg and Al, extracted with 1 mol L^-1^ KCl; H+Al: potential acidity at pH 7.0, extracted with 0.5 mol L^-1^ calcium acetate; SB: sum of bases; CEC_(t):_ effective cation exchange capacity; CEC_(T):_ total cation exchange capacity; V: base saturation; m: aluminum saturation; P-rem: remaining phosphorus.

**Fig 2:**
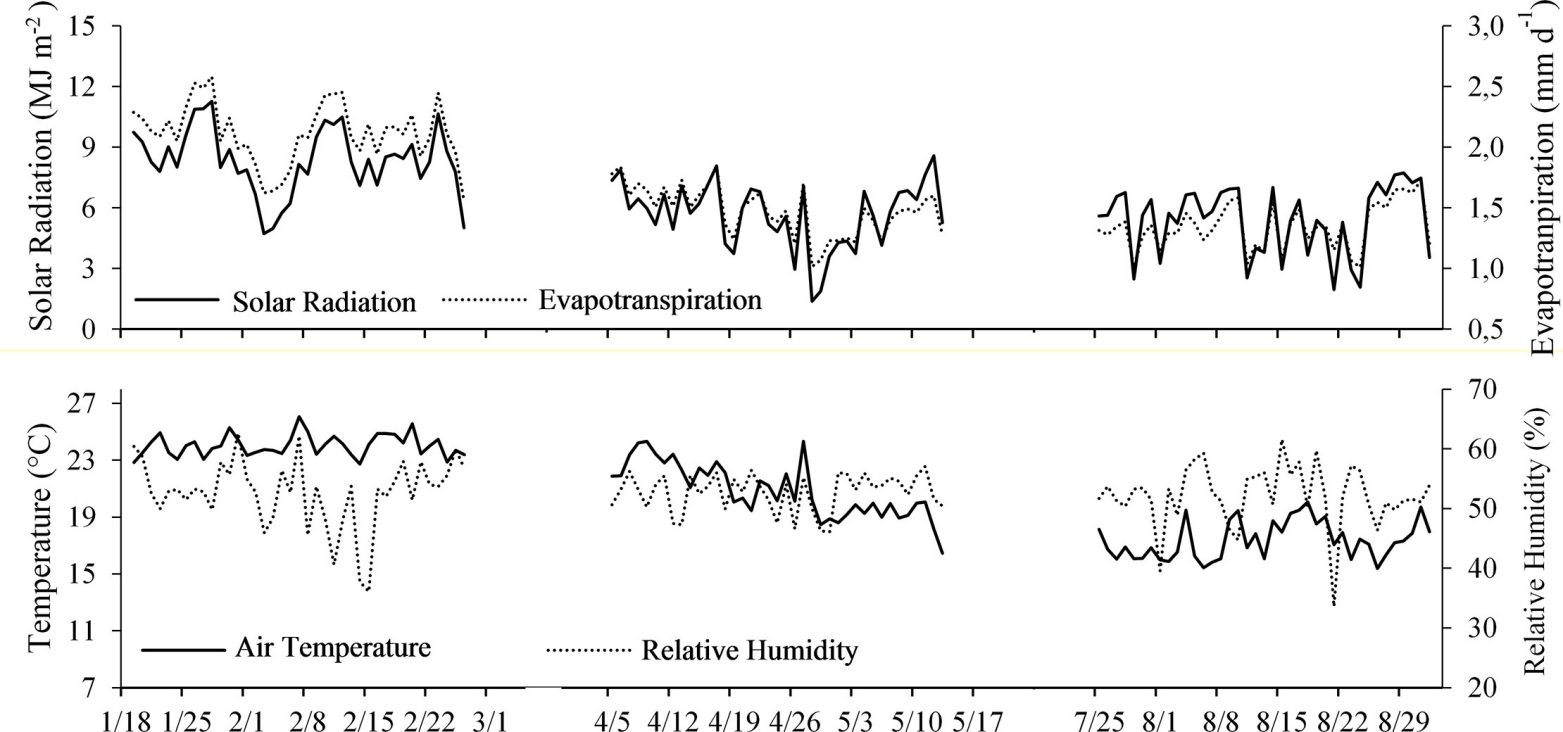
Daily variation of solar radiation, reference evapotranspiration, temperature and relative humidity for the three cycles of lettuce cultivation in Viçosa-MG in 2017.

The sowing of lettuce was carried out in polystyrene trays, with transplanting of the seedlings to the beds (in small depressions spaced 0.25 m between rows and 0.25 m between plants) when they had four definitive leaves (30 days after sowing). Each sampling unit (Fig 2) had an area of 1 m^2^ (1 m in length and 1 m in width), consisting of 16 plants, and the four central plants were evaluated.

The evaluations were made 39 days after seedling transplantation in three cultivation cycles (01/19 to 02/26/2017—cycle 1, 04/05 to 05/13/2017—cycle 2 and 07/25 to 01/09/2017—cycle 3). A randomized block design (RBD) was used with four replications in a split plot scheme. Five irrigation depths (50, 75, 100, 125 and 150% of the irrigation depth for crop evapotranspiration replacement—ETc) were implemented in the plots, and four lettuce cultivars (Raider Plus—Americana type; Luara—Lisa type; Vanda—Crespa type; Imperial—Roxa type) were planted in the subplots (Fig 1). Therefore, data from 80 observations (5 depths × 4 replicates × 4 plants sampled) were used to evaluate each analyzed characteristic, each cultivar and each cycle.

A drip irrigation system (Fig 1) was used, with lateral lines composed of drip tape (Amanco brand) 16 mm in diameter and 15 thousandths of inch thick. The spacing between the drip tapes was 0.50 m, which allowed for the irrigation of two rows of plants. The emitters (drippers), spaced 20 cm apart, operated at a service pressure of 98 kPa (~ 10 mca), applying an average flow of 1.8 L h^-1^.

Manual weeding was performed weekly, and there were no pests and diseases capable of causing damage to the lettuce quality or yield.

### Irrigation management

The irrigation system was evaluated according to [19], modified by [20], collecting the dripper flows at eight points along the lateral line and in four lateral lines along the line of bypass. The distribution uniformity coefficient (DUC) was calculated by Eq 1:

$$DUC=\frac{q_{25}}{q_{m}}$$ (1)

where q_25_ is the average of the lowest quartile of flows (L h^-1^) and q_m_ is the average flow (L h^-1^).

The application efficiency was considered 100%, and the average DUC was 93.1%. Thus, the irrigation efficiency (E_i_) was 93.1% for all three cycles. To maintain pressure uniformity during the irrigation period (beyond this research), two lateral lines were always simultaneously kept open, such as the practical example outlined in S1 Table.

The real irrigation required (RIR_LOC_) and total irrigation required (TIR_LOC_) in the localized systems (mm) for 100% ETc treatment were estimated using Eqs 2 and 3, respectively, adapted from [21].

$${\mathrm{RIR}}_{\mathrm{LOC}}=\sum_{\mathrm{day}\;1}^{i}{\mathrm{ET}}_{0}\;K_{C}\;K_{S}\;K_{L}‐C$$ (2)

$${TIR}_{LOC}=\frac{{RIR}_{LOC}}{E_{i}}$$ (3)

where ET_0_ is the reference evapotranspiration (mm day^-1^), K_C_ is the coefficient of culture (dimensionless), K_S_ is the coefficient of soil water depletion (dimensionless), K_L_ is the location coefficient (dimensionless), and C is the constant relative to the elevation of the water table (mm).

ET_0_ was estimated according to Penman-Monteith FAO-56 [22] by Eq 4, with a wind speed equal to 0.2 m s^-1^ (recommended for protecting the environment).

$${\mathrm{ET}}_{0}=\frac{0,408\;s(R_{N}‐G)+\gamma \frac{900}{t+273}U_{2}\frac{\left(e_{s}‐e\right)}{10}}{s+\gamma (1+0,34\;U_{2})}$$ (4)

where s is the slope of the saturation pressure curve versus temperature (kPa°C^-1^), R_N_ is the radiation balance (MJ m^2^ day^-1^), G is the soil heat flux (MJ m^2^ day^-1^), γ is the psychrometric constant (kPa°C^-1^), t is the average air temperature (°C), U2 is the wind speed at 2 m in height (m s^-1^), e_s_ is the water vapor saturation pressure (hPa), and e is the current water vapor pressure (hPa).

Kc values were adopted from [23] and [24] for each stage (phase) of crop development: phase I (Kc = 0.65)—from transplant until the crop covers 10% of the land surface; phase II (Kc = 0.85)—from the end of the first phase to the effective full coverage of the ground surface; and phase III (Kc = 1.00)—from the end of the second phase until the maximum vegetative development, when lettuce is harvested. K_S_ and K_L_ were calculated according to [21] and [25] by Eqs 5 and 6, respectively:

$$K_{S}=\frac{\mathrm{Ln}(\mathrm{CWB}+1)}{\mathrm{Ln}(TWC+1)}$$ (5)

$$K_{L}=\frac{P}{100}+0,15\left(1-\frac{P}{100}\right)$$ (6)

where CWB is the current water depth (mm), TWC is the total water capacity (mm), and P is the highest percentage value between the wet and shaded area (%).

Over five days prior to beginning climate irrigation management, soil water content was monitored at a depth of 0–0.20 m using tensiometers based on the soil water retention curve θ = 0.1813 + (0,3443 − 0.1813)/[1 + (0.01|Ψ_m_|)^2^]^0.49^ and by the direct stove method to ensure soil moisture at field capacity. Irrigation depths were differentiated for 7 days after seedling transplantation (acclimatization period of the seedlings to the soil).

### Weather data

Meteorological data (Fig 2) were obtained through the automatic meteorological station E 4000 (IRRIPLUS) centrally installed in the protected environment (Fig 1). Daily reference evapotranspiration (ET_0_) was estimated from the data of solar radiation (Rs), maximum and minimum temperatures [and with these values the average air temperature (T_average_)] and relative humidity (RH).

The average daily Rs decreased over the crop cycles, ranging from 4.7 to 11.3 MJ m^-2^ in the first cycle, from 1.4 to 8.6 MJ m^-2^ in the second, and from 2.0 to 7.7 MJ m^-2^ in the third (Fig 2). This behavior influenced the decrease in T_average_ and ET_0_. The T_average_ varied from 22.7 to 26.1°C in the first cycle, from 16.5 to 24.3°C in the second cycle, and from 15.4 to 20.1°C in the third cycle. The average RH ranged from 36.1 to 62.6%, from 46.1 to 57.0% and from 33.6 to 61.6% in cycles 1, 2 and 3, respectively.

The total accumulated degrees per day for each crop cycle were obtained according to [26] and [27] and were determined as 546.9, 423.5 and 287.6°C day^-1^ for cycles 1, 2 and 3, respectively.

ET_0_ presented average values of 2.1 mm day^-1^ (ranging from 1.6 to 2.6 mm day^-1^) in the first cycle, 1.5 mm day^-1^ (from 1.0 to 1.8 mm day^-1^) in the second cycle, and 1.3 mm day^-1^ (from 1.0 to 1.7 mm day^-1^) in the third cycle. The ET_0_ that occurred during each cycle was used to determine the irrigation depth of 100% ETc applied in the treatments (Table 2).

**Table 2:** Real and total irrigation required in each treatment and lettuce-growing cycle.

Cycle	Irrigation required	Irrigation depths (% of ETc)				
		50	75	100	125	150
1	Real (mm)	28.5	42.8	57.0	71.3	85.5
	Total (mm)	30.6	45.9	61.2	76.5	91.8
2	Real (mm)	19.1	28.6	38.1	47.6	57.2
	Total (mm)	20.5	30.7	40.9	51.2	61.4
3	Real (mm)	18.1	27.2	36.2	45.3	54.3
	Total (mm)	19.4	29.2	38.9	48.6	58.3

Due to the absence of rainfall inside the protected environment, a high frequency of irrigation (2-day irrigation shifts) was required, maintaining the soil with moisture near the field capacity and with little water requirement to reach the total storage at each irrigation period (Table 2).

### Characteristics analyzed

- **Leaves:** *relative content of chlorophylls (SPAD index) A (Chl-A) and B (Chl-B) and total chlorophyll (Chl-T)* were expressed as indirect in vivo measurements with a portable chlorophyll meter SPAD-502 (Soil Plant Analysis Development, Minolta Camera Co., Osaka, Japan) in SPAD values (higher values of SPAD indicate a higher content of chlorophyll [28]) from in intact and freshly mature leaves at four points on the leaf and two centimeters from the margin, always at 12 o'clock; the *number of commercial leaves (NCL*, *units per plant)* were expressed as the number of marketable leaves per plant; the *number of noncommercial leaves (NNCL*, *units per plant)* were expressed as the number of damaged or unlabeled leaves per plant; *leaf length and width (LL and LW*, *cm)* were measured as the greatest length and width of the more developed leaves; *the leaf area index (LAI*, *dimensionless*) was measured by the leaf discs method, using 30 discs of 2.0 cm in diameter (3.14 cm^2^) per plant taken from the basal, median and apical portions of three leaves, avoiding the sampling of the central vein, similar to [29]; the *fresh leaf mass (FLM*, *g)* was expressed as the mass of all leaves per plant; *the dry leaf mass (DLM g)* was expressed as the mass of all leaves per plant, dried in a forced ventilation oven at 65°C until reaching constant mass.
- **Roots:** *the fresh root mass (FRM*, *g) and dry root mass (DRM*, *g) per plant* were obtained as outlined above for the fresh and dry leaf masses*; the root length (RL*, *cm)* was obtained for the longest root per plant, measured with a ruler; *the root volume (RV*, *cm*^*3*^*) per plant* was measured by the displacement volume of water inside a graduated cylinder.
- **Stems:** *the fresh stem mass (FSM*, *g)* was determined as the fresh shoot mass without leaves; *the stem length (SL*, *cm)* was determined using a ruler; *the stem average diameter (SMD*, *mm)* was expressed as the average of three measurements (base, middle and tip) using a pachymeter; *the stem volume (SV*, *cm*^*3*^*)* was measured by the displacement volume of water inside a graduated cylinder.
- **Aerial Part:** *the aerial part height (APH*, *cm)* was measured as the distance from the soil surface to the top of the shoot, measured just before harvest; *the aerial part diameter (APD*, *cm)* was obtained by taking two measurements of the diameter of each plant with a ruler, one in the horizontal plane and one in the perpendicular direction; *the aerial part volume (APV*, *cm*^*3*^*)* was measured by the displacement volume of water inside a graduated cylinder of known dimensions; *the aerial part total mass (APTM*, *g)* was expressed as the sum of the commercial and noncommercial masses per plant; *the aerial part commercial mass (APCM*, *g)* was measured as the mass of the marketable aerial part; *the mass of the aerial part loss at harvest (MLH*, *g) per plant* was determined by subtracting the commercial mass from the total aerial part mas*s; the crop yield (Y*, *kg m*^*-2*^*)* was an estimation of the ratio between the production of the useful plot (based on APCM) and the area occupied by it, extrapolating to an area of 1 m^2^; *the water productivity (WP*, *kg m*^*^-3^*^*)* (water use efficiency by area) was expressed as the ratio between *Y* (kg m^-2^) and the amount of water applied (mm) per treatment.

### Statistical analysis

The obtained data were subjected to analysis of variance at 5% and 1% probability by the F test. When significant at 5%, the effects of depth were subjected to regression analysis, and the effects of cultivars were compared by the Tukey test at 5% probability. For regression analysis, the linear, quadratic, cubic, square root, potential, exponential, hyperbolic, logarithmic, cubic root, log-log, Ln-Ln and Exp(x) models were tested. The best model was obtained considering the significance of the F test for the regression equation, up to a 5% probability, the coefficient of determination (R2), and the representativeness of biological behavior by the equation. The curve inflection points were obtained by the first-order partial derivative of the respective regression equations. Data were subjected to principal component analysis (PCA). We used GENES and SISVAR software for statistical analysis.

## Results and discussion

The coefficients of variation (CV%) from the analysis of variance (Tables 3, 4 and 5) for the three crop cycles ranged from 5.4% to 45.0% in the plot (average of 25%) and from 4.7% to 5.0% in the subplot (average of 27%). The highest CV values occurred for MLH, APV and LAI, which are directly linked to fresh phytomass, especially of the leaves. Lettuce has, on average, 94% water in its constitution, and therefore, part of the inherent variability of this culture occurs due to variations of the water content from one plant to another, increasing the CV of the experiment [30].

**Table 3:** Average squares, significance of the F test (ANOVA) and average values for leaf characteristics: relative content of chlorophylls A (Chl-A) and B (Chl-B) and of total chlorophyll (Chl-T), number of commercial leaves (NCL), number of noncommercial leaves (NNCL), leaf length and width (LL and LW), leaf area index (LAI), fresh leaf mass (FLM), and dry leaf mass (DLM g) as a function of different cultivars and irrigation depths in three lettuce crop cycles.

Charact.	Cycle	Average Square—F Test			Irrigation Depth (% ETc)	Lettuce Cultivars							
		ID	LC	ID x LC		Raider Plus		Luara		Vanda		Imperial Roxa	
Chl-A (%)	1	5.9E+0 ^ns^	1.2E+2**	1.4E+1 ^ns^		24.7	A	20.4	B	18.9	B	20.6	AB
	2	6.9E+0 ^ns^	5.1E+1 ^ns^	5.3E+0 ^ns^		ȳ = 20.9							
	3	4.0E+0 ^ns^	1.5E+2**	2.9E+0 ^ns^		22.8	A	16.8	B	21.4	A	22.0	A
Chl-B (%)	1	1.8E+0 ^ns^	2.4E+1**	2.7E+0 ^ns^		6.9	A	4.9	B	4.4	B	5.2	B
	2	9.3E-1 ^ns^	8.3E+0 ^ns^	8.6E-1 ^ns^		ȳ = 5.3							
	3	1.9E+0 ^ns^	9.2E+1**	1.4E+0 ^ns^		6.5	A	3.7	B	2.5	C	7.0	A
Chl-T (%)	1	1.2E+1 ^ns^	2.5E+2**	2.7E+1 ^ns^		31.6	A	25.3	B	23.3	B	25.8	B
	2	1.2E+1 ^ns^	1.0E+2 ^ns^	8.9E+0 ^ns^		ȳ = 26.2							
	3	1.0E+1 ^ns^	3.6E+2**	6.8E+0 ^ns^		29.3	A	20.5	C	23.9	B	29.0	A
NCL (ud pl^-1^)	1	8.4E+0 ^ns^	1.9E+3**	1.3E+1 ^ns^		16.5	C	37.8	A	19.1	BC	20.4	B
	2	2.1E+1 ^ns^	3.6E+3**	4.8E+1*	50%	26.3	B	41.5	A	28.5	B	-	
					75%	22.8	B	47.8	A	30.3	B	-	
					100%	22.0	B	54.0	A	28.0	B	-	
					125%	24.5	B	50.3	A	30.8	B	-	
					150%	21.8	C	52.5	A	31.0	B	-	
	3	1.2E+1 ^ns^	9.0E+2**	1.5E+0 ^ns^		18.7	B	27.7	A	15.7	C	12.1	D
NNCL (ud pl^-1^)	1	2.3E+0 ^ns^	2.9E+1**	3.6E-1 ^ns^		6.2	A	5.5	A	3.6	B	4.1	B
	2	9.1E+0 ^ns^	6.0E+1**	4.5E+0 ^ns^		5.9	B	9.3	A	7.3	B	-	
	3	7.2E-1 ^ns^	7.4E+1**	5.3E-1 ^ns^		2.0	C	5.9	A	3.8	B	1.7	C
LL (cm)	1	2.9E+0 ^ns^	8.4E+1**	2.6E+0 ^ns^		17.7	C	22.3	A	21.7	AB	20.7	B
	2	8.5E+0 ^ns^	3.2E+1**	2.2E+0 ^ns^		22.2	AB	21.1	B	23.6	A	-	
	3	2.4E+0 ^ns^	2.3E+2**	2.7E+0 ^ns^		25.3	A	21.8	B	25.8	A	18.5	C
LW (cm)	1	7.4E+0 ^ns^	1.4E+2**	4.2E+0 ^ns^		15.5	C	14.3	C	18.5	B	20.1	A
	2	1.3E+1 ^ns^	1.8E+2**	1.2E+1 ^ns^		18.7	A	13.6	B	18.9	A	-	
	3	1.7E+0 ^ns^	4.1E+2**	2.5E+0 ^ns^		25.7	A	14.8	C	19.2	B	19.2	B
LAI (Dimensionless)	1	1.6E+1 ^ns^	2.4E+2**	1.5E+1 ^ns^		16.1	A	14.3	A	12.5	A	7.9	B
	2	3.6E+1 ^ns^	1.6E+1	1.3E+2*	50%	26.9	A	11.9	B	13.0	B	-	
					75%	20.5	A	12.5	A	20.1	A	-	
					100%	15.3	A	21.8	A	14.5	A	-	
					125%	11.7	A	21.0	A	14.7	A	-	
					150%	11.6	A	14.1	A	14.8	A	-	
	3	2.6E+1 ^ns^	3.1E+3**	3.7E+1 ^ns^		9.7	C	32.1	A	20.4	B	3.7	D
FLM (g)	1	2.3E+3 ^ns^	3.8E+4**	1.6E+3 ^ns^		236.4	A	182.4	B	172.7	B	130.1	C
	2	6.3E+3*	1.4E+4**	3.2E+3*	50%	222.1	A	137.8	B	178.1	AB	-	
					75%	227.8	A	183.0	A	201.8	A	-	
					100%	223.8	AB	257.3	A	180.0	B	-	
					125%	273.0	A	216.5	A	225.3	A	-	
					150%	271.2	A	226.6	AB	182.8	B	-	
	3	1.9E+3 ^ns^	1.1E+5**	6.1E+2 ^ns^		265.3	A	161.3	B	137.9	B	89.9	C
DLM (g)	1	3.8E+0 ^ns^	7.8E+1**	5.8E+0 ^ns^		10.9	A	10.0	A	10.0	A	6.4	B
	2	1.9E+1*	3.1E+1**	8.4E+0 ^ns^		12.1	B	13.5	AB	14.6	A	-	
	3	2.9E+0 ^ns^	3.9E+1**	2.1E+0 ^ns^		13.9	A	13.6	A	11.2	B	11.4	B

Charact.: evaluated characteristic; ID: irrigation depth; LC: lettuce cultivar; ID x LC: interaction between ID and LC; ETc: crop evapotranspiration

* and **: significance at 5% and 1% of probability, respectively, by the F test

^ns^: not significant; Averages followed by the same letter in the line do not differ by Tukey's test (p <0.05).

**Table 4:** Average square significance of the F test (ANOVA) and average values for characteristics of roots and stems: fresh root mass (FRM), dry root mass (DRM), root length (RL), root volume (RV), fresh stem mass (FSM), stem length (SL), stem average diameter (SMD) and stem volume (SV) as a function of different cultivars and irrigation depths in three lettuce crop cycles.

Charact.	Cycle	Average Square—F Test			Irrigation Depth (% ETc)	Lettuce Cultivars							
		ID	LC	ID x LC		Raider Plus		Luara		Vanda		Imperial Roxa	
FRM (g)	2	2.9E+0 ^ns^	1.1E+2**	4.1E+0 ^ns^		6.6	C	11.2	A	9.5	B	-	
	3	6.0E+0 ^ns^	5.7E+2**	2.6E+0 ^ns^		8.4	B	15.5	A	14.1	A	3.9	C
DRM (g)	2	3.3E-2 ^ns^	7.8E+0**	8.7E-2 ^ns^		0.8	A	1.2	A	1.1	A	-	
RL (cm)	2	9.4E-1 ^ns^	6.8E+0*	1.4E+0 ^ns^		13.1	A	12.6	AB	12.0	B	-	
	3	3.7E-1 ^ns^	6.9E+1**	8.2E+0 ^ns^		11.8	C	13.7	BC	16.2	A	14.6	AB
RV (cm^3^)	2	2.5E+0 ^ns^	9.9E+1*	3.4E+0 ^ns^		5.8	C	10.2	A	8.1	B	-	
	3	7.6E+0 ^ns^	4.8E+2**	3.2E+0 ^ns^		7.2	C	14.2	A	12.1	B	3.3	D
FSM (g)	1	2.6E+2 ^ns^	3.3E+3**	1.9E+2 ^ns^		26.2	C	56.4	A	44.4	B	47.8	AB
	2	9.8E+2**	8.9E+3**	3.8E+2**	50%	16.5	B	34.0	A	43.0	A	-	
					75%	21.4	B	42.3	A	55.3	A	-	
					100%	19.8	C	73.3	A	53.3	B	-	
					125%	18.3	B	69.3	A	72.3	A	-	
					150%	25.9	B	56.5	A	68.4	A	-	
	3	3.3E+1 ^ns^	1.0E+3**	1.4E+1 ^ns^		6.0	C	13.4	B	19.7	A	4.2	C
SL (cm)	1	2.0E+1 ^ns^	8.2E+2**	1.8E+1 ^ns^		11.0	C	19.0	B	20.1	B	26.6	A
	2	5.0E+1**	9.5E+2**	2.1E+1 ^ns^		7.8	C	17.1	B	21.2	A	-	
	3	1.1E+1 ^ns^	4.9E+3**	1.2E+1 ^ns^		29.9	A	4.0	B	5.5	B	33.6	A
SMD (mm)	1	5.4E+0 ^ns^	5.8E+1**	4.4E+0 ^ns^		16.1	A	17.1	A	14.0	B	13.4	B
	2	1.4E+1*	1.5E+1**	8.8E+0**	50%	15.3	A	16.2	A	17.4	A	-	
					75%	17.3	A	18.1	A	18.3	A	-	
					100%	17.3	B	22.6	A	17.0	B	-	
					125%	17.8	A	19.8	A	18.3	A	-	
					150%	18.9	A	18.5	A	18.6	A	-	
	3	5.8E+0 ^ns^	1.8E+2**	3.3E+0 ^ns^		13.0	C	15.5	B	17.8	A	10.9	D
SV (cm3)	1	2.5E+2 ^ns^	2.8E+3**	1.6E+2 ^ns^		25.4	B	52.7	A	43.2	A	47.1	A
	2	8.6E+2**	8.5E+3**	6.2E+2**	50%	22.0	A	36.3	A	43.6	A	-	
					75%	22.4	B	43.0	AB	55.4	A	-	
					100%	20.4	C	77.0	A	42.8	B	-	
					125%	19.2	B	73.4	A	72.1	A	-	
					150%	24.5	B	59.9	A	70.5	A	-	
	3	1.6E+1 ^ns^	7.1E+2**	1.2E+1 ^ns^		5.6	B	6.1	B	16.8	A	3.6	B

Charact.: evaluated characteristic; ID: irrigation depth; LC: lettuce cultivar; ID x LC: interaction between ID and LC; ETc: crop evapotranspiration

* and **: significance at 5% and 1% of probability, respectively, by the F test

^ns^: not significant; Averages followed by the same letter in the line do not differ by Tukey's test (p <0.05).

**Table 5:** Average square significance of the F test (ANOVA) and average values for aerial part characteristics: aerial part height (APH), diameter (APD) and volume (APV), aerial part total mass (APTM) and commercial mass (APCM), mass of aerial part loss at harvest (MLH), crop yield (Y) and water productivity (WP) as a function of different cultivars and irrigation depths in three lettuce crop cycles.

Charact.	Cycle	Average Square—F Test			Irrigation Depth (% ETc)	Lettuce Cultivars								
		ID	LC	ID x LC		Raider Plus		Luara		Vanda		ImperialRoxa		
APH (cm)	1	4.1E+1*	9.0E+2**	1.2E+1 ^ns^		21.8	C	33.2	B	33.7	B	37.2	A
	2	5.2E+1**	6.6E+2**	1.2E+1 ^ns^		17.6	B	26.7	A	28.2	A	-	
	3	1.8E+1 ^ns^	4.0E+2**	8.6E+0 ^ns^		20.2	B	24.7	A	26.6	A	16.7	C
APD (cm)	1	1.7E+0 ^ns^	2.3E+2**	1.4E+0 ^ns^		22.8	C	30.5	A	29.3	B	28.9	B
	2	5.4E+1**	1.5E+2**	2.7E+1*	50%	14.8	B	27.5	A	25.0	A	-	
					75%	20.0	B	26.8	A	24.0	AB	-	
					100%	23.0	A	26.3	A	26.0	A	-	
					125%	25.8	A	29.5	A	27.0	A	-	
					150%	27.0	A	27.3	A	26.3	A	-	
	3	1.5E+1 ^ns^	1.3E+2**	4.8E+0 ^ns^		25.8	B	29.0	A	28.5	A	23.6	C
APV (cm3)	1	5.7E+4 ^ns^	2.2E+5**	3.5E+4 ^ns^		614.4	A	615.3	A	470.5	B	407.7	B
	2	1.2E+5 ^ns^	1.7E+5*	5.3E+4 ^ns^		656.8	AB	664.2	A	501.8	B		
	3	8.8E+3 ^ns^	2.4E+5**	4.7E+3 ^ns^		403.5	A	270.8	B	227.8	BC	137.7	C
APTM (g)	1	5.3E+3^ns^	6.6E+4**	3.3E+3 ^ns^		338.9	A	261.9	B	246.1	B	200.6	C
	2	1.6E+4**	2.8E+3 ^ns^	4.5E+3 ^ns^		ȳ = 296.9								
	3	3.0E+3 ^ns^	1.2E+5**	9.4E+2 ^ns^		287.5	A	203.8	B	188.5	B	97.3	C
APCM (g)	1	3.8E+3 ^ns^	2.6E+4**	2.7E+3 ^ns^		262.5	A	238.8	AB	217.1	B	177.9	C
	2	1.2E+4**	6.0E+2 ^ns^	4.5E+3*	50%	238.5	A	172.1	A	225.0	A	-	
					75%	249.2	A	225.3	A	254.5	A	-	
					100%	243.5	B	330.6	A	239.0	B	-	
					125%	291.3	A	285.8	A	292.3	A	-	
					150%	297.0	A	283.1	A	254.3	A	-	
	3	2.2E+3 ^ns^	1.1E+5**	8.0E+2 ^ns^		271.3	A	174.6	B	157.6	B	92.4	C
MLH (g)	1	2.1E+2 ^ns^	1.3E+4**	1.5E+2 ^ns^		76.3	A	23.1	B	29.1	B	22.6	B
	2	5.8E+2 ^ns^	1.6E+3**	1.2E+2 ^ns^		46.2	A	28.6	B	39.5	AB	-	
	3	1.4E+2 ^ns^	3.0E+3**	7.4E+1 ^ns^		16.2	B	29.2	A	30.9	A	4.9	C
Y (kg m^-2^)	1	4.9E+7 ^ns^	3.4E+8**	3.5E+7 ^ns^		3.0	A	2.7	AB	2.5	B	2.0	C
	2	1.5E+8**	7.8E+6 ^ns^	5.8E+7*	50%	2.7	A	2.0	A	2.6	A	-	
					75%	2.9	A	2.6	A	2.9	A	-	
					100%	2.8	B	3.8	A	2.7	B	-	
					125%	3.3	A	3.3	A	3.3	A	-	
					150%	3.4	A	3.2	A	2.9	A	-	
	3	2.9E+7 ^ns^	1.4E+9**	1.0E+7 ^ns^		3.1	A	2.0	B	1.8	B	1.1	C
WP (kg m^-3^)	1	1.3E+4**	2.9E+3**	4.4E+2 ^ns^		84.5	A	75.9	AB	69.0	B	56.3	C
	2	1.8E+4**	3.2E+2 ^ns^	1.5E+3**	50%	200.4	A	144.5	B	189.0	A	-	
					75%	139.6	A	126.2	A	142.5	A	-	
					100%	102.3	AB	138.9	A	100.4	B	-	
					125%	97.9	A	83.8	A	98.2	A	-	
					150%	83.2	A	90.3	A	71.2	A	-	
	3	2.6E+4**	2.9E+4**	1.6E+3*	50%	249.7	A	159.6	B	135.5	B	76.6	C
					75%	144.4	A	93.0	B	75.3	B	55.1	B
					100%	110.7	A	72.6	AB	69.3	AB	46.4	B
					125%	100.8	A	60.5	AB	63.1	AB	32.6	B
					150%	86.0	A	58.4	AB	50.3	AB	24.8	B

Charact.: evaluated characteristic; ID: irrigation depth; LC: lettuce cultivar; ID x LC: interaction between ID and LC; ETc: crop evapotranspiration

* and **: significance at 5% and 1% of probability, respectively, by the F test

^ns^: not significant; Averages followed by the same letter in the line do not differ by Tukey's test (p <0.05).

Tables 3, 4 and 5 show that there were interactions between irrigation depth and lettuce cultivars only for cycle 2 (APCm^2^, Y2, WP2, WP3, APD2, NCL2, FLm^2^, LAI2, FSm^2^, SMD2, and SV2) and for cycle 3 (WP3). A significant effect for lettuce cultivars, as a variation factor, was observed in almost all evaluated characteristics for the three crop cycles. Irrigation depth had a significant effect on APTMT2, WP1, APH1, APH2, DLm^2^ and SL2.

The existence of a linear relationship between the number of leaves and the air temperature, accumulated through the degrees per day (GD) thermal summation, has confirmed that temperature is the main factor controlling the rate of leaf emission [27]. However, in the present study, the highest number of leaves per plant for all cultivars tested (Table 3) occurred in cycle 2, in which the average temperature was lower than that of cycle 1 (Fig 2). The lower incidence of radiation in cycle 2 (Fig 2) induced an increase in the number of leaves in the plant, increasing the amount of chlorophyll and consequent maintenance of the photosynthetic rate required for the plant.

The Raider Plus cultivar better adapt to environments with a lower incidence of light, similar to the protected environment used in this study, since it presented a higher percentage of chlorophylls A and B and total chlorophyll (Table 3) than that of the other cultivars and, consequently, carried out more photosynthesis. A higher concentration of chlorophyll increased the generation of leaf quantity (higher NCL and NNCL), leading to relatively high phytomass values (FLM and DLM). Leaf quality (higher LL and LW) was also influenced when the plants had higher leaf areas (LL and LW) and, consequently, a higher specific leaf area [31] increased the LAI of the Raider Plus cultivar, which also propagated greater phytomass of the plants [32].

The quality and freshness of commercially sold vegetables can be monitored by measuring the chlorophyll content [28]. According to [33], changes in total chlorophyll concentration relative to the photosynthetic rate may follow an opposite-to-expected pattern, exhibiting higher values under reduced radiation intensities. This increase in pigment content in shaded leaves has been attributed to the increase in the number and size of the chloroplasts, the amount of chlorophyll produced by chloroplasts and/or better development of the *Grana* within the chloroplasts. This effect is considered an efficient adaptive process of the acclimatization of plants to environments with low light intensity [34].

This behavior benefited the development of the Raider Plus cultivar, intensifying the green coloration and increasing its visual quality, which promotes greater attractiveness and commercialization in relation to the other cultivars.

The Luara cultivar also showed high values of chlorophyll (Table 3), but its NCL values were the most prominent in all crop cycles, independent of the applied irrigation depth, which reflected in larger aerial parts (APD and APV) (Table 5). According to [35], a higher NCL increases the quality of lettuce, adding value to the crop during its commercialization, and this characteristic is often linked to genetic factors.

Better photosynthetic characteristics, for both the Raider Plus and Luara cultivars, promoted better root contribution (greater FRM, DRM, and RL), with a higher volume of explored soil (RV), reducing water stress of the plant even under smaller applied irrigation depths (50% and 75% of ETc) (Table 4). The optimal root characteristics possibly generated higher nutrient absorption and better soil exploration, increasing the amount and quality of leaves in the plant and therefore facilitating photosynthesis [36]. The increase in leaf quantity and quality provided higher LAI values (Table 3), with fast closure of the plant stand and less competition with unwanted plants, with further development of the Luara cultivar [37].

Although the Vanda cultivar had higher values of some of the characteristics, such as LAI, LL, FRM, DRM, RL, RV and APD (Tables 3, 4 and 5), higher values of stem characteristics [FSM, SMD, SV, and mainly SL (plant weeding)] and lower values of leaf characteristics reduce the visual and commercial quality of this cultivar compared to the others. The Imperial Roxa cultivar was less adapted to the conditions studied since it presented lower values in almost all characteristics, except for SL, an undesirable characteristic in lettuce commercialization. Different species, under water stress, may present survival mechanisms, including variations in the stomatal responses, osmotic adjustment and greater movement of photoassimilates to the roots [38], with metabolic energy consumption, which may affect the plant’s production and commercial quality [9].

As described previously, the plant physiological factors of the Raider Plus and Luara cultivars were better than those of the Vanda and Imperial Roxa cultivars. The plant physiological factors were influenced in a directly proportional way in production (APTM and APCM), as well as in Y. The Raider Plus and Luara cultivars, with larger numbers of leaves (NCL and NNCL), smaller stems (lower SL, SMD and SV) and higher volumes (higher APV), presented higher APTM and APCM values, which are directly proportional to Y.

Although the Raider Plus cultivar presented high crop loss indexes (MLH), APTM was enough to confer higher APCM and higher Y for this crop. The highest yield average of the three cycles was observed for the Raider Plus cultivar (3.0 kg m^-2^), followed by the Luara (2.8 kg m^-2^), Vanda (2.7 kg m^-2^) and the Imperial Roxa (1.5 kg m^-2^) cultivars.

The highest values of Y observed in the present study for the Raider Plus, Luara and Vanda cultivars were similar to the maximum value (3.6 kg m^-2^) presented by [39] for conventional lettuce cultivation in soil.

**Fig 3:**
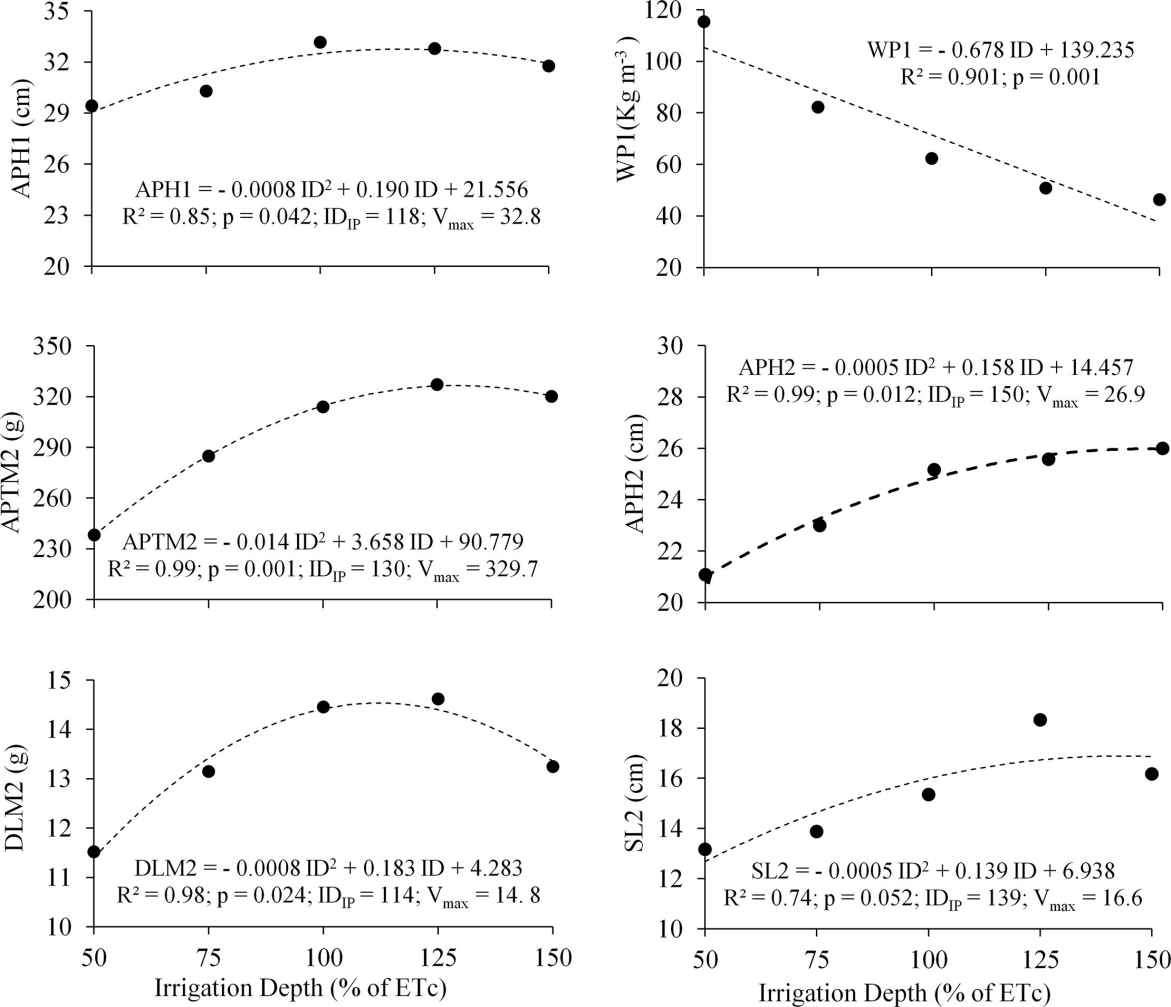
Average values of the aerial part height (APH) and water productivity (WP) of cycle 1, and the aerial part total mass (APTM), aerial part height (APH), dry leaf mass (DLM) and stem length (SL) of cycle 2 as a function of irrigation depths (ID) for lettuce cultivars. The number after the variable abbreviation indicates the cultivation cycle. ID_IP_: irrigation depth referring to the inflection point of the curve.

Fig 3 presents the average behavior of the agronomic characteristics of the four cultivars that did not show significant interaction at a 5% probability by the F test between the cultivars studied and the irrigation depths, with variable maximum values and an irrigation depth corresponding to the curve inflexion point (ID_IP_).

An approximate average value of water replacement depth equal to 110% ETc provides water conditions for the lettuce crop to express its greatest potential in important variables (APTM and DLM), with PA near to the average (Fig 3).

**Fig 4:**
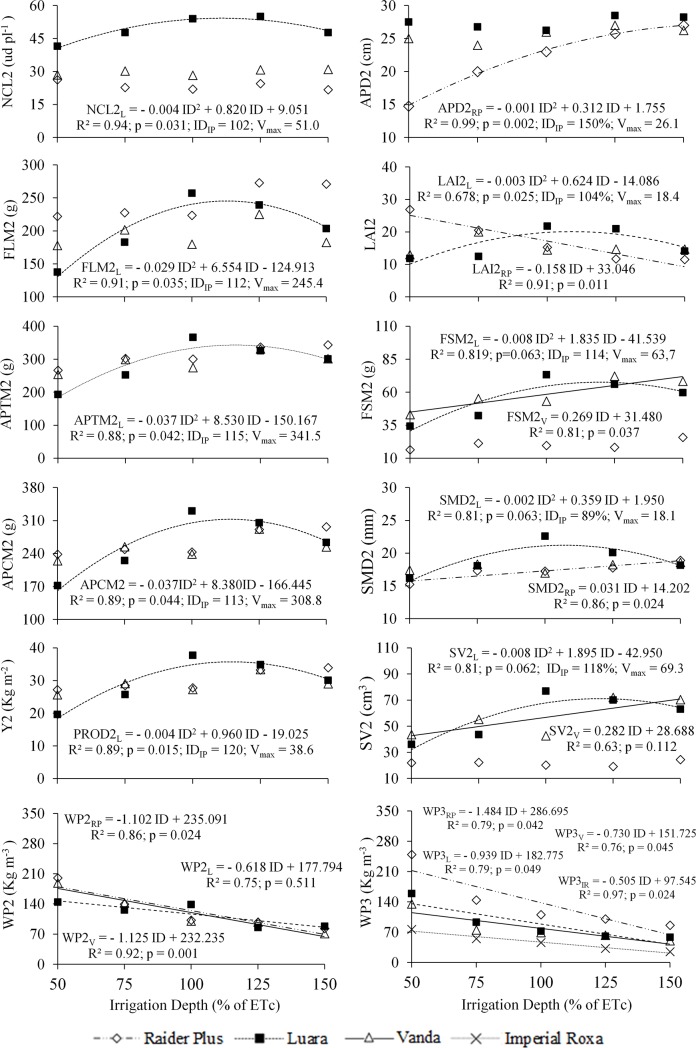
Average values of the number of commercial leaves (NCL), aerial part diameter (APD), fresh leaf mass (FLM), leaf area index (LAI), fresh stem mass (FSM), stem average diameter (SMD), stem volume (SV), aerial part total mass (APTM), aerial part commercial mass (APCM), and crop yield (Y) of cycle 2 and the water productivity (WP) of cycles 2 and 3 as a function of irrigation depths (ID) for lettuce cultivars. The number after the variable abbreviation indicates the cultivation cycle. ID_IP_: irrigation depth referring to the inflection point of the curve.

Interactions between the studied cultivars, as a function of the irrigation depth and referring to the maximum point of the curve inflection point (ID_IP_) obtained by deriving the respective equations, are shown in Fig 4. Note that trend lines were used to highlight only the cultivars that were statistically influenced (5% probability by the F test) by the variation depths, but the averages of the other cultivars were also inserted (Fig 4).

The Luara cultivar was the most influenced by irrigation depth (Fig 4), explained by second-degree polynomial equations for the characteristics NCL2, FLm^2^, LAI2, APTm^2^, APCm^2^, Y2, FSm^2^, SMD2, and SV2 and by linear equations for WP in cycles 2 and 3. The Vanda cultivar was the second most influenced by irrigation depth, adjusted by linear equations for FSm^2^, SMD2, SV2, and WP in cycles 2 and 3. The Raider Plus cultivar was slightly affected by irrigation depth, as explained by the second-degree polynomial equation for APD2, LAI2 and WP in the last two cycles. Finally, the Imperial Roxa cultivar showed an irrigation depth influence only for WP in the third cycle.

Although higher water productivity (WP) was observed for the lowest irrigation depth used (50% of ETc), similar to [9], important variables, such as NCL and FLM, reached maximum values by applying an average depth of approximately 110% of ETc (Fig 4).

Note that the optimal aerial part characteristics, which directly influence Y, were obtained by irrigation depths higher than 100% water replenishment. Possibly, the global efficiency of the system did not achieve 100% efficiency in water absorption because of losses by percolation, redistribution of water in the soil and areas with a water deficit [40]. Another explanation for this fact is the potential soil salinity caused by the high salt levels (Table 1) accumulated in the soil used in the present research.

**Fig 5:**
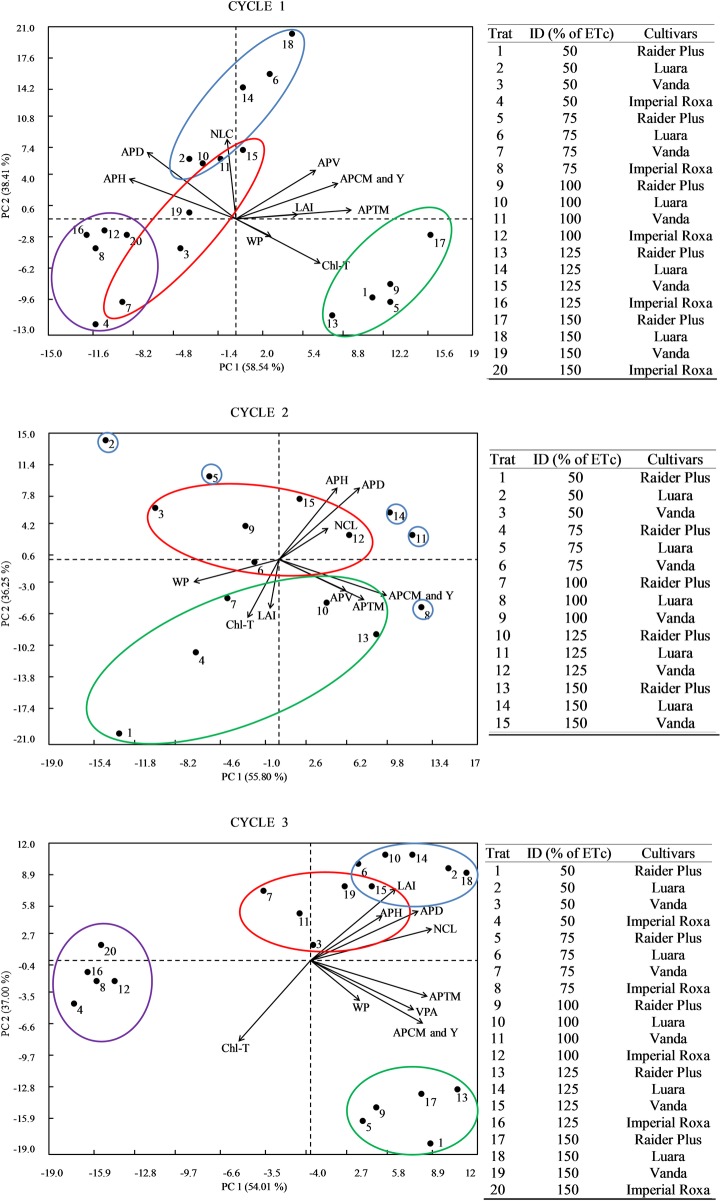
Principal component analysis (PCA) for the aerial part total mass (APTM), aerial part commercial mass (APCM); mass of aerial part loss upon harvesting (MLH), crop yield (Y), water productivity (WP), aerial part height (APH), aerial part diameter (APD), aerial part volume (APV), leaf length (LL), leaf width (LW), fresh leaf mass (FLM), dry leaf mass (DLM), leaf area index (LAI), fresh stem mass (FSM), stem length (SL), stem average diameter (SMD), stem volume (SV) and content of total chlorophyll (Chl-T) in cycles 1, 2 and 3 under different irrigation depths for lettuce cultivars. Trat: Treatment. ID: irrigation depth. Circles: green (Raider Plus); blue (Luara); red (Vanda); and purple (Imperial Purple). Viçosa-MG, DEA-UFV, 2017.

The principal component analysis (PCA) is represented in Fig 5. The first major component (PC1) explains a large portion of the parameter variances (agronomic characteristics). The second main component (PC2) explains a smaller part of said variance but more than that of the last component (coincident with the number of parameters evaluated), which explains little or almost nothing of the existing variance. Usually, only the first two components are graphically represented (Fig 5) because together, they generally represent more than 80% of this variance [41, 42, 43].

Comparatively, the closer that a treatment is plotted to a given characteristic, the more suitable this treatment will be to obtain higher values of that characteristic.

Treatments involving the Raider Plus cultivar (1, 5, 9, 13 and 17), regardless of the irrigation depth, were concentrated on the right of the central axis (Fig 5). This behavior of Raider Plus occurred mainly in the first and third cycles, with intense association with variables that positively influence the quality and productivity of the lettuce, such as Chl-T, LAI and Y (first cycle), Chl-T, LAI and WP (second cycle), and APV, Y and WP (third cycle). Treatments involving the Luara cultivar (Fig 5) favored the NCL and APD variables in the first cycle (treatments 2, 10 and 14), NCL, APV and Y in the second cycle (treatments 8 and 11), and APD, APH and NCL in the third cycle (treatments 2, 6, 10, 14 and 18).

Unlike the Raider Plus and Luara cultivars, the Vanda and Imperial Roxa cultivars concentrated their treatments on the negative side of the CP1 axis (Fig 5), in the opposite direction of the important variables for lettuce quality, confirming their nonpreference for the important variables. The situation of the Imperial Roxa cultivar was even more removed, with reduced association with the variables of interest for lettuce production.

The angle between the direction of any two vectors (Fig 5) indicates the association amplitude between the variables represented by these vectors, and it is inversely proportional with the correlation; that is, the smaller the angle between vectors referring to the characteristics, the greater the correlation degree between them. Thus, Y, as the main characteristic, was positively influenced by the APV, APCM, APTM, LAI, and Chl-T characteristics in the first cycle, by APV, APCM and APTM in the second cycle, and by LAI, APD, APH, NCL, APTM and APCM in the third cycle. In contrast, Y was negatively influenced by APH and APD in cycle 1 and by Chl-T in cycles 2 and 3.

## Conclusions

The Raider Plus and Luara cultivars can be recommended for the cultivation of lettuce in protected environments, with blue plastic covering, in the Zona da Mata of Minas Gerais State and in regions with similar conditions.

Lettuce irrigation should be performed with a depth to restore 110% of the crop evapotranspiration to the region under conditions similar to those of the current study.

Future research comparing the same cultivars and irrigation depths in protected environments with different coverings is encouraged by the authors and may provide different conclusions than that of the current research.

## Supporting information

S1 Fig Research localization (Viçosa-MG, Brazil). (TIF)

S1 Table Practical example of opening sideline valves. (TIF)
