# Supplementary figures and images for: Agronomic performance of lettuce cultivars submitted to different irrigation depths
Source: PLoS One. 2019 Dec 11;14(12):e0224264. doi: 10.1371/journal.pone.0224264 (PMC6905512; doi:10.1371/journal.pone.0224264)

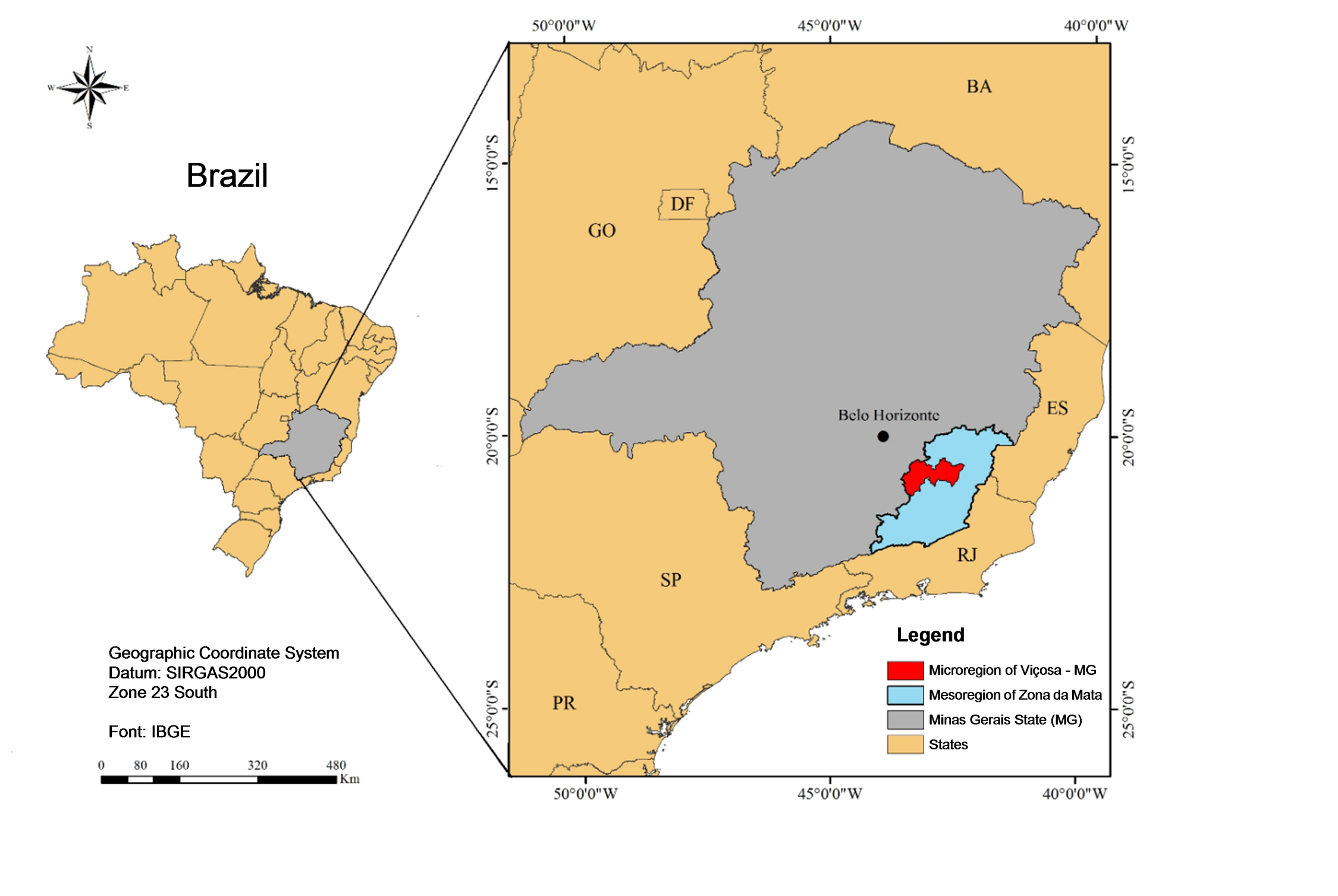

Supplement: S1 Fig — (TIF) [file pone.0224264.s001.tif]

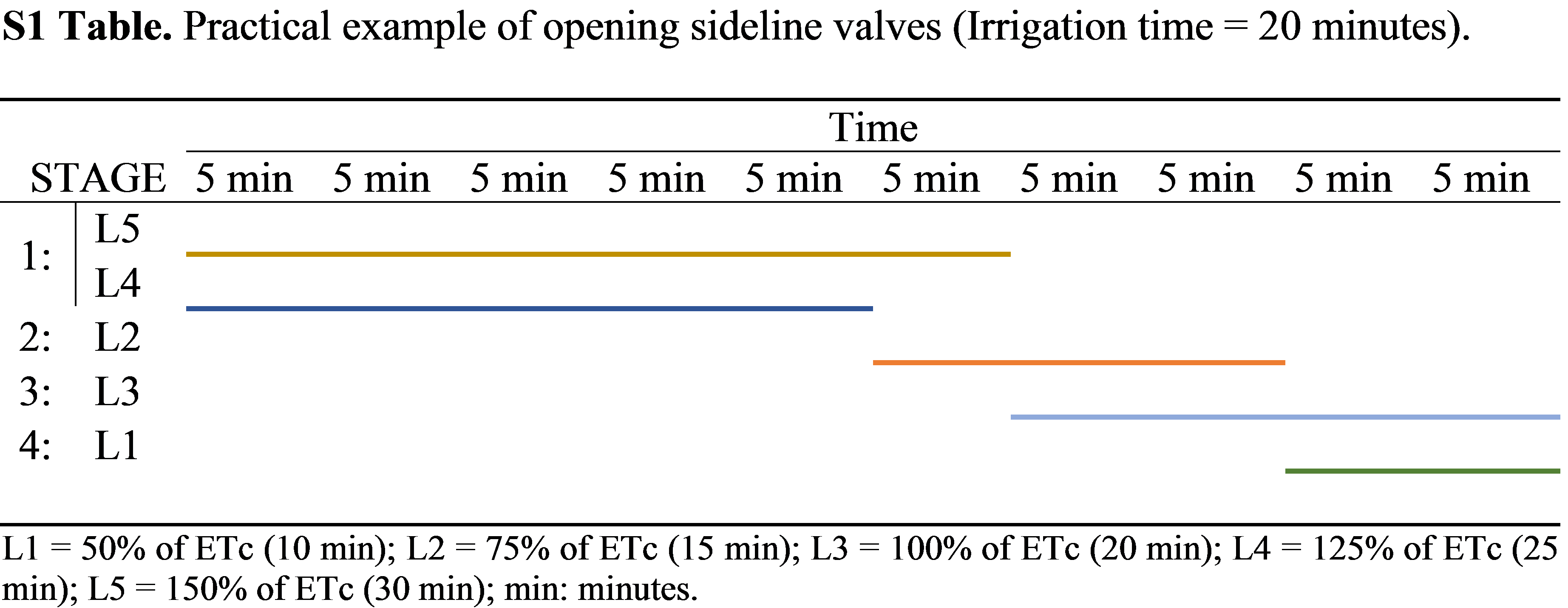

Supplement: S1 Table — (TIF) [file pone.0224264.s002.tif]
